# Patient-reported outcomes in terms of swallowing and quality of life after prophylactic versus reactive percutaneous endoscopic gastrostomy tube placement in advanced oropharyngeal cancer patients treated with definitive chemo-radiotherapy: Swall PEG study

**DOI:** 10.1186/s13063-022-06991-6

**Published:** 2022-12-21

**Authors:** Tatiana Dragan, André Van Gossum, Frederic Duprez, Yassine Lalami, Yolene Lefebvre, Sofiana Mootassim-Billah, Sylvie Beauvois, Akos Gulyban, Christophe Vandekerkhove, Petra Boegner, Marianne Paesmans, Lieveke Ameye, Antoine Digonnet, Marie Quiriny, Didier Dequanter, Samuel Lipski, Esther Willemse, Alejandra Rodriguez, Sebastien Carlot, Yasemin Karaca, Marc Lemort, Patrick Emonts, Clémence Al Wardi, Dirk Van Gestel

**Affiliations:** 1 Department of Radiation Oncology (Head and Neck Unit), Institut Jules Bordet, Université Libre de Bruxelles, 1 rue Héger Bordet, 1000 Brussels, Belgium; 2 Consultant at the Department of Gastroenterology and Clinical Nutrition, Hopital Erasme and Institut Jules Bordet, Brussels, Belgium; 3 Department of Radiotherapy-Oncology, Universitair Ziekenhuis Gent, Gent, Belgium; 4 Medical Oncology Clinic, Institut Jules Bordet, Université Libre de Bruxelles, Brussels, Belgium; 5 Department of Radiology, Institut Jules Bordet, Université Libre de Bruxelles, Brussels, Belgium; 6 Department of Radiation Oncology, Speech Therapy, Institut Jules Bordet, Université Libre de Bruxelles, Brussels, Belgium; 7 Medical Physics Department, CHU Saint-Pierre, Université Libre de Bruxelles, Brussels, Belgium; 8 Medical Oncology Clinic, CHU de Bruxelles, CHU Saint-Pierre, Université Libre de Bruxelles, Brussels, Belgium; 9 Data Centre, Institut Jules Bordet, Université Libre de Bruxelles, Brussels, Belgium; 10 Department of Head and Neck Surgery, Institut Jules Bordet, Université Libre de Bruxelles, Brussels, Belgium; 11 Department of Otorhinolaryngology and Head and Neck Surgery, CHU de Bruxelles, CHU Saint-Pierre, Université Libre de Bruxelles, Brussels, Belgium; 12 Department of Otorhinolaryngology and Head and Neck Surgery, Erasmus Hospital, Université Libre de Bruxelles, Brussels, Belgium; 13 Stomatology, Oral and Maxillofacial Surgery Department, Erasmus Hospital, Université Libre de Bruxelles, Brussels, Belgium; 14 Department of Radiation Oncology, Institut Jules Bordet, Université Libre de Bruxelles, Brussels, Belgium

**Keywords:** Oropharyngeal squamous cell carcinoma, Head and neck cancer, Endoscopic gastrostomy, Radiotherapy, Patient-reported outcome

## Abstract

**Background:**

Percutaneous endoscopic gastrostomy (PEG) is often used to provide nutritional support in locally advanced head and neck cancer patients undergoing multimodality treatment. However, there is little published data on the impact of prophylactic versus reactive PEG. PEG placement may affect swallowing-related physiology, function, and quality of life. The Swall PEG study is a randomized controlled phase III trial testing the impact of prophylactic versus reactive PEG on patient-reported outcomes in terms of swallowing and quality of life in oropharyngeal cancer patients.

**Methods:**

Patients with locally advanced oropharyngeal cancer receiving chemo-radiotherapy will be randomized to either the prophylactic or reactive PEG tube group. Randomization will be stratified by human papillomavirus (HPV) status and unilateral versus bilateral positive neck lymph nodes. The primary objective of the study is the patient’s reported outcome in terms of swallowing (MD Anderson Dysphagia Inventory (MDADI)) at 6 months. Secondary objectives include health-related quality of life, dosimetric parameters associated with patient-reported outcomes, chemo-radiation toxicities, PEG tube placement complications, the impact of nutritional status on survival and toxicity outcomes, loco-regional control, overall survival, the impact of HPV and tobacco smoking on survival outcomes and toxicities, and the cost-effectiveness of each treatment strategy.

**Discussion:**

Findings from this study will enhance clinical evidence regarding nutritional management in oropharyngeal cancer patients treated by concurrent chemo-radiation.

**Trial registration:**

ClinicalTrials.gov NCT04019548, study protocol version 2.0_08/08/2019. Registered on 15 July 2019

## Background

Oropharyngeal squamous cell carcinoma (OPSCC), the most common malignancy among the head and neck sites, arise in the tonsils, pharyngeal wall, soft palate, base of tongue, and vallecula. Around 98,412 new cases were diagnosed worldwide in 2020 resulting in 48,143 deaths [1].

### Treatment

The treatment of OPSCC has evolved over time, especially since the discovery of the HPV entity. Although a monotherapy of either surgery or radiation therapy (RT) remains the main treatment modality for early-stage OPSCC, concurrent chemoradiation therapy (CCRT) has largely replaced RT alone for locally advanced disease. The use of CCRT and intensified RT was implemented widely in the late 1990s and early 2000s [2–6]. Based on four large randomized trials, conventionally fractionated external beam RT with concurrent administration of three cycles of high-dose cisplatin (100 mg/m^2^) given once every 3 weeks represents the current standard in definitive and adjuvant treatment of locally advanced head and neck squamous cell carcinoma (HNSCC), as it results in significantly better locoregional control (LRC) and overall survival (OS) relative to RT alone [4, 5, 7, 8]. However, the toxicity of this approach is a serious limitation which precludes further treatment intensification and thereby further improvement in outcome [9].

### Treatment-related side effects and patient-reported outcomes

With the increasing recognition of the side effects of modern combined modality treatment for HNSCCs on the one hand and its increased cure rates (particular for HPV-initiated oropharyngeal cancers) on the other hand, it is important to measure and reduce the late treatment-related toxicity. Evaluation of side effects and patient-reported outcomes (PROs) have been developed empirically for over more than 30 years. At the same time multiple grading scales dating from the nineties such as World Health Organization (WHO) scale; Radiation Therapy Oncology Group/European Organization for Research and treatment of Cancer (RTOG/EORTC); Late Effects Normal Tissue Task Force (LENT)—Subjective, Objective, Management, Analytic (SOMA) scales; and Common Terminology Criteria for Adverse Events (CTCAE) are used in parallel to “objectively” report on treatment-related side effects [10]. Each system uses slightly different criteria, with some focused on symptoms and others on interventions, leading to potential discrepancies when scoring toxicities in different systems. The CTCAE scale is widely accepted as the standard classification and severity grading scale for adverse events in clinical trials including multimodality approach. Furthermore, the CTCAE scale is periodically revised, with Version 5.0 (November 27, 2017) being the latest for now. However, this scale is not very detailed on RT toxicity. On the other hand, PROs, defined as a health outcome directly reported by the patient, cover the *subjective* domain of the side effects. Exploratory work suggests that patient-reported outcome measurements (PROMs) can be used with a high degree of subject engagement and compliance [11]. These Quality of life (QOL) questionnaires measure a subjective concept, reflecting a subject’s sense of well-being, incorporating domains such as physical, functional, social, and emotional well-being. Their development and use are based on the science of psychometrics [12]. The EORTC QOL-HN35 questionnaire, specifically developed for HNSCCs patients, was validated in the 1990s and is nowadays used as a worldwide standard [13]. Its major advantage is the symptom severity of HNSCCs and its treatment-specific adverse effects (e.g., swallowing problems, xerostomia and changes in speech, etc.) being assessed by the subject. The EORTC QOL group conducted two systematic reviews concluding that the extensive use of HN35 module throughout the world has demonstrated robust psychometric features in different languages [14, 15]. However, some reported methodological problems suggested that the instrument could be improved in some areas, while studies investigating targeted and/or multimodal therapy consider some QOL issues specific to these treatments not to be covered by the HN35 module. Therefore, the module has been adapted into the EORTC QOL-HN43, of which the final validation (phase IV) has been completed in February 2017 [16]. In order to assess the swallowing function, one of the validated instruments is MD Anderson Dysphagia Inventory (MDADI). It is a self-administered questionnaire evaluating the impact of dysphagia on the QOL of patients with head and neck cancer [17].

### Swallowing-related outcomes and the effect of prophylactic PEG tube placement

In HNSCC treatment, swallowing is one of the most important toxicities in terms of QOL [18]. Langendijk et al. conducted a study with assessments by the EORTC/RTOG toxicity scale and the EORTC QLQ 30 and HN35 modules at 6, 12, 18, and 24 months after the end of RT. All patients (*n* = 425) were treated by RT alone (3D conformal without attempts to spare the salivary glands) or in combination with chemotherapy and/or surgery for HNSCCs of the oral cavity, oropharynx, nasopharynx, hypopharynx, and larynx. They concluded a higher toxicity score to be correlated with significant impairment of QOL in general. The degree of xerostomia and dysphagia had a significantly greater impact on QOL compared to other toxicities as late reactions of the skin, subcutaneous tissue, mucous membrane, and larynx, with xerostomia being more frequent while dysphagia having the bigger impact. In order to prevent malnutrition and dehydration following these frequent side effects, some centers perform percutaneous endoscopic gastrostomy (PEG) tubes placed prophylactically (pPEG). According to recent ESPEN guidelines, enteral feeding is strongly recommended in patients with obstructing head and neck cancer with expected severe radiation-induced oral mucositis. However, the effect of pPEG on clinical outcome has not been investigated in randomized trial [19]. A recent research has suggested that pPEG use may negatively affect swallowing physiology, function, and/or QOL, especially in the long term [20]. The evaluation of swallowing function was performed with various subject- and physician-rated instruments, and there is still a lack of data from clinical trials systematically collecting information on swallowing via instrumental objective measurements (e.g., video fluoroscopic swallowing examination, manometry). The available data regarding the frequency and severity of dysphagia and swallowing-related outcomes vary and are inconclusive; hence, the impact of pPEG use on swallowing and swallowing-related outcomes remains unclear [21]. The choice between prophylactic and reactive placement of a PEG tube is generally based on the experience of an individual institution rather than on a high level of evidence [22, 23]. A prospective randomized trial is the most robust way to answer the question on which modality provides better functional outcome for HNSCC patients.

## Methods/design

### Study design

This is a multi-institutional prospective, randomized, phase III clinical trial evaluating PROs on swallowing using the MDADI, 6 months after the end of CCRT in patients randomized to either the pPEG tube group or the reactive PEG tube group.

**Fig. 1:**
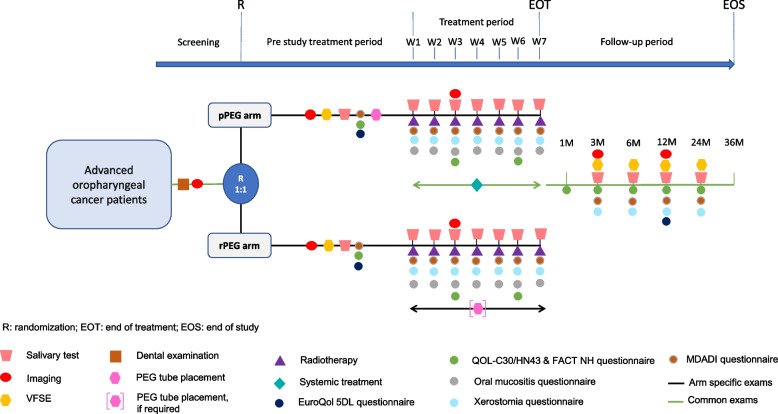
Study design

The study design is illustrated in Fig. 1.

### Assignment of intervention

#### Sequence generation

The patients will be randomly assigned at the Institut Jules Bordet with a 1:1 allocation as per a computer-generated randomization schedule stratified by institution, HPV status (negative, positive), and lymph node metastasis (no lymph node metastasis or unilateral lymph node metastasis vs bilateral lymph node metastasis).

#### Concealment mechanism

The use of a web-based allocation system and the minimization process will ensure that the allocation sequence is concealed until the intervention is assigned.

#### Implementation

Patients who give consent to participation and fulfil the inclusion criteria will be randomized. For this, Head and Neck Cancer teams will contact the local clinical study coordinator who will schedule the baseline assessment. Randomization will occur after the baseline assessment is completed. The clinical study coordinator is responsible for communicating the allocated group to the clinician who will inform the participant.

### Study objectives and statistical analyses

The primary goal of this study is to assess and analyze the PROs in terms of swallowing (MDADI) at 6 months after the end of CCRT in subjects randomized to either the pPEG tube group or the rPEG tube group.

The secondary objectives are as follows:

- Health-related QOL (HRQOL; EORTC QLQ-C30; EORTC QLQ-H&N43 module) and Functional Assessment of Cancer Therapy-Head and Neck (FACT-HN).
- Dosimetric parameters associated with PROs.
- CCRT-related toxicities and PEG tube placement complications.
- The impact of nutritional status on survival and toxicity outcomes.
- Clinical and radiological tumor response.
- Loco-regional control (LRC), distant recurrence (DR), second primaries (SP), disease-free survival (DFS), disease-specific survival (DSS), and overall survival (OS). Time to event variable will be assessed from the day of randomization to the day of the first record of any of the considered events.
- The impact of HPV and tobacco smoking on these secondary endpoints.
- Cost-effectiveness of each treatment strategy.
- Clinical validation of cancer prediction models available at www.predictcancer.org.

### Sample size calculation

The registered subjects are randomized (1:1) between pPEG and rPEG tube placement. The randomization will be stratified by the HPV status and unilateral versus bilateral metastatic neck lymph nodes. The MDADI is a patient-reported outcome scale, and it is composed of 19 items: emotional (6 questions), functional (5 questions), and physical (8 questions) scored from 1 to 5 plus one global question. The mean score of the 19 items will be multiplied by a factor of 20 to obtain a MDADI total score, which ranges from 20 (extremely low functioning) to 100 (high functioning). A difference of 10 points are considered as the minimal clinically important difference (MCID) in the MDADI, as it corresponds to the difference observed between patients taking soft food versus tube feeds [24, 25]. The study’s aim is to detect a difference of at least 10 points between the two arms in the MDADI composite score. Considering an average MDADI composite score of 50 in the prophylactic arm and 60 in the reactive arm (standard deviation 17.5), 100 subjects are needed to detect a significant difference between the two arms (power of 0.80, alpha 0.05 two-sided test). Taking a 10% drop-out rate into account, a total of 110 randomized subjects will be required. To assess survival data, we will use Kaplan-Meier curves with log-rank test. Cox’s proportional hazard model will be used for calculating hazard ratios. To compare continuous outcome variables between the two arms, *t*-test or Wilcoxon test will be used. Categorical variables between the two arms will be analyzed with the chi-square test or Fisher exact test. No data imputation of missing values will be performed. Stratified analyses by HPV status (negative, positive) will be performed as exploratory analyses.

### Patients’ characteristics

Newly diagnosed, histologically confirmed primary OPSCC patients, candidate for curative intent RT and systemic treatment, will be included in the study. The patients for this study are recruited in the Jules Bordet Institute, Centre Hospitalier Universitaire Saint- Pierre and Hôpital Erasme—Clinique Universitaire de Bruxelles. Inclusion and exclusion criteria are listed in Table 1.

**Table 1:** Study inclusion, exclusion, and drop out criteria

**Inclusion criteria**	
• Age ≥ 18 years old	
• ECOG performance status ≤ 2	
• Newly diagnosed, histologically confirmed primary, stage III or IV HPV-negative^a^ oropharyngeal cancer, or T3-4/ N0-3 and T0-4/N1-3 HPV-positive oropharyngeal cancer according to the UICC TNM 8th edition • Candidate for curative intent CCRT	
• No prior or current anticancer treatment for the HNSCC (e.g., neo-adjuvant chemotherapy, surgery)	
• No contraindication for cisplatin-based chemotherapy	
• Ability to understand and complete the questionnaires (language proficiency, cognitive functioning as judged by principal investigator upon screening)	
**Exclusion criteria**	
• Severe malnutrition at baseline according to the Global Leadership Initiative on Malnutrition (GLIM) criteria [26]	
• Dysphagia requiring a liquid or puree texture-modified diet (grade ≥ 2 (CTCAE_v.5)	
• Distant metastasis	
• Serious coagulation disorders (INR > 1.5, PTT > 50s, platelets ≤ 100,000/μL or 100 × 10^9^/L)	
• Other malignancies in the 3 years prior to study entry except for surgically cured carcinoma in situ of the cervix, in situ breast cancer, incidental finding of stage T1a or T1b prostate cancer, and cured basal/squamous cell carcinoma of the skin	
**Drop out criteria**	
• Refusal to participate	

*ECOG* Eastern Cooperative Oncology Group, *GLIM* Global Leadership Initiative on Malnutrition, *CTCAE* Common Terminology Criteria for Adverse Events

^a^HPV tumor status testing is performed by surrogate marker p16 immunohistochemistry either on the primary tumor or from cervical nodal metastases. The threshold for positivity is at least 70% nuclear and cytoplasmic expression with at least moderate to strong intensity [27]. Additional HPV-specific testing are permitted for the protocol

### Study interventions

#### Percutaneous endoscopic gastrostomy

In the pPEG arm, the PEG tube will be placed before the start of the study treatment (CCRT). The enteral nutrition will start following the assessment by the clinical dietitian in order to complete the current oral consumption according to the estimated energy needs based on 30 to 35 kcal/kg adapted and 1.2 to 1.5 g proteins/kg body weight (BW), with an adaptation when required. A rPEG tube will be placed and enteral nutrition initiated in case of a decrease in the oral intake of less than two thirds of the estimated energy requirements based on 30–35 kcal/adapted kg BW and 1.2–1.5 g proteins/adapted kg BW, for a period of/or anticipated to be, greater than 7 days or a weight loss ≥ 5% of pre-treatment baseline. A nutritional counseling by a dedicated dietitian will be offered at all study time points. The nutritional status will be assessed according to the GLIM criteria [26].

#### Speech therapy

All patients will be encouraged to maintain oral intake as much as possible during treatment and as long as it remains safe to do so as advised by the speech pathologist. Prophylactic swallowing exercises for all patients will be presented prior to the start of treatment, and patients will be instructed to start these specific swallowing exercises from day one of treatment and to continue for the duration of their CCRT. All patients will receive 30-min assisted speech therapy once a week to assure the successful achievement of the exercises at home. They will be asked to perform at least 2 non-assisted exercise sessions per day. Each single exercise should be repeated at least 10 times, and a total session duration will last for at least 15 min. Written instructions will be given to the patients. In order to check the motivation and adherence to therapy, patients will be asked to fill out a diary to note which exercises they really performed in different categories (swallowing maneuvers, lips or tongue movements, etc.) and the frequency per day. Exercises too difficult to practice alone will only be performed during assisted training sessions.

#### Patient-reported outcomes

**Fig. 2:**
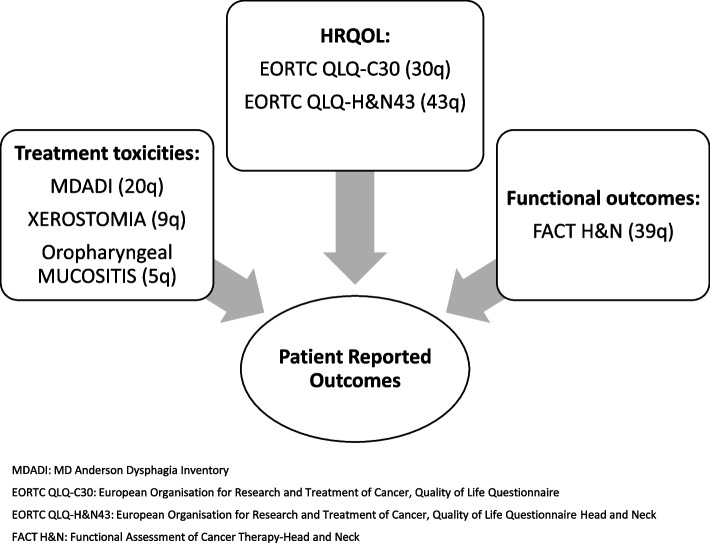
Patient-reported outcomes

PROs will be measured at different time points of the study via the questionnaires illustrated in Fig. 2. They will be completed at the hospital before the clinical examination, using CANKADO software, class I medical device, within the European Union (registration number DE/CA59/11976/2017) version 1.6 30-DEC-2020, installed on an electronic portable device. A paper version of the questionnaires will be proposed to any patient unfamiliar with electronic devices.

### Treatment

All patients will be treated with a concurrent regimen of cisplatin-based chemotherapy and slightly accelerated simultaneous integrated boost (SIB) intensity-modulated RT (IMRT). In 32 fractions (5 fr per week), a 69.12-Gy (2.16 Gy/fr) will be administered to the high-risk planning target volume (PTV) and 56 Gy (1.75 Gy/fr) to the elective PTV using IMRT/volumetric modulated arc therapy (VMAT) with 6 MV photons. The gross target volume (GTV), clinical target volume (CTV), and organs at risk (OARs) will be delineated by a specialized head and neck radiation oncologist according to well-defined and uniform guidelines [28–31]. Well-defined dose-volume constraints will be used for the treatment planning (Table 2). Treatment plans will be created with an Elekta’s Monaco treatment planning system, version 5.51.2.

**Table 2:** Dose-volume constraints

	D95%^**a**^	Dnear-min (D98%)	D5%	Median dose (D50%)	Soft constraints	Hard constraints
PTV_69.12	≥ 65.55 Gy	≥ 62.2 Gy	73.8 Gy	69.12 Gy		
PTV_56	≥ 53.2 Gy	≥ 50.4 Gy		56 Gy ± 2%		
Spinal cord					D0.1% < 45 Gy	D0.1 < 48 Gy
PRV spinal cord					D1% < 50 Gy	D5 < 50 Gy
Brain stem					D0.1% < 50 Gy	D5 < 50 Gy
PRV brain stem					D1% < 60 Gy	D5 < 60 Gy
Ipsilateral parotid					D50% < 25 Gy	
Contralateral parotid					D50% < 20 Gy	
Pharyngeal constrictor muscles					D50% < 40 Gy	
Supraglottic larynx					D50% < 45 Gy	
Glottis					D50% < 20 Gy	
Cricopharyngeal muscle					D0.1% < 60 Gy	
Cervical esophagus					D50% < 30 Gy	
Extended oral cavity-PTV					D50% < 35 Gy	
Mandible					D0.1% < 70 Gy	

^a^*Dx%* dose in *x*% of the volume

Maximal measures will be taken to avoid prolonging the overall treatment time. In case of machine breakdown or holidays, an extra fraction should be performed within the same week, with at least 6 h between fractions. However, no more than six 2.16-Gy fractions will be delivered per week. Two concurrent chemotherapy regimens are allowed: cisplatin 100 mg/m^2^ IV (days 1 and 22) and weekly cisplatin 40 mg/m^2^ IV (days 1, 8, 15, 22, 29, and 36).

### Toxicity evaluation

A clinical assessment will be performed weekly, during the radiation therapy and at 1, 3, 6, 12, 24, and 36 months after the treatment. Concurrently, patients will pursue the standard follow-up visits: every 2–3 months for the first 2 years, every 3–6 months in 3rd, 4th, and 5th years and then yearly further on. Toxicity is defined as acute when occurring during RT and/or in the 3 months after the end of RT and as late from then on. Intensity of all adverse events will be graded by a specialized head and neck radiation oncologist using the National Cancer Institute (NCI)–CTCAE version 5.0 on a 5-point scale (grades 1 to 5) and the RTOG/EORTC grading scale [32].

As an objective evaluation of dysphagia, a video fluoroscopic swallowing examination (VFSE) will be performed at baseline, 3, 6, 12, and 24 months after the treatment. A salivary test analyzing saliva quantity and quality will evaluate xerostomia at defined study time points illustrated in Fig. 1.

All serious adverse events (SAEs) related to a protocol-mandated intervention will be reported on SAE for and transmitted to the EudraVigilance Clinical Trial Module, the Competent Authorities, the Ethics Committees, and the participating investigators.

### Clinical outcome evaluation

Response assessment will be based on clinical examination, dual-energy CT scan, and PET-CT scan at 3 and 12 months following the end of RT. Classification of early response will be made in accordance with the Response Evaluation Criteria In Solid Tumors (RECIST) [33].

#### Ancillary and post-trial care

If any patient suffers a complication or harm from trial participation, the clinical team will provide appropriate medical and surgical care as per institutional standards. The participant centers will continue to provide post-trial care and long-term follow-up for all patients participating in the trial.

#### Data monitoring

In order to ensure the quality assurance of the trial the clinical research physician assisted by study coordinator performs the medical review meetings on a regular basis. The study team is assisted in case of problems with patient evaluation: safety, eligibility, and treatment compliance. For data processing and management, the electronic data capture system OpenClinica will be used. The study coordinator will undertake regular monitoring. Observations and findings will be documented and made available to the coordinating investigator and local study site.

## Discussion

The incidence of OPSCC in Europe is expected to increase with changes in disease characteristics as younger patients and more curable disease. A better understanding of toxicities from different treatment modalities will permit more appropriate decisions for the patient with better long-term quality of life. In this regard, PROMs may lead to a better capture of the patient’s experience and might enhance toxicity awareness by clinicians. This article describes the protocol of a randomized controlled trial to evaluate the impact of the prophylactic versus reactive gastrostomy tube placement on PROs in OPSCC patients, by combining subjective and objective measures. The PROMs will be analyzed and compared with dosimetrical and clinical data. Although pPEG will help to maintain a correct nutritional status by a faster initiation of enteral or mixed enteral and oral feeding, these patients have a tendency to abandon oral feeding more quickly with a potential detrimental effect on the anatomical swallowing structures, resulting in late dysphagia. Alternatively, a close dietician follow-up with reactive gastrostomy placement could be a more appropriate practice with less long-term patient-reported dysphagia and better QOL. The Swall PEG study will improve the healthcare quality enhancing the scientific evidence in the management of the OPSCC patient.

## Trial status

Protocol version number and date: V2.0, dated 8 August 2019. The first participant was enrolled on 11 December 2019.

## Protocol amendments

The protocol amendments are submitted for approval to the Committee of Ethics and the regulatory authority for approval. All study staff receive notice of changes by study coordinator.

## Data Availability

The datasets used and analyzed during the current study will be available from the corresponding author on reasonable request.

## Abbreviations

PEG: Percutaneous endoscopic gastrostomy
pPEG: Prophylactic percutaneous endoscopic gastrostomy
rPEG: Reactive percutaneous endoscopic gastrostomy
MDADI: MD Anderson Dysphagia Inventory
HPV: Human papillomavirus
OPSCC: Oropharyngeal squamous cell carcinoma
RT: Radiotherapy
CCRT: Concurrent chemoradiotherapy
HNSCC: Head and neck squamous cell carcinoma
LRC: Loco-regional control
OS: Overall survival
PROs: Patient-reported outcome
WHO: World Health Organization
RTOG: Radiation Therapy Oncology Group
EORTC: European Organization for Research and treatment of Cancer
LENT-SOMA: Late Effects Normal Tissue—Subjective, Objective, Management, Analytic SOMA
CTCAE: Common Terminology Criteria for Adverse Events
PROMs: Patient-reported outcome measurements
QOL: Quality of life
DR: Disease recurrence
DP: Disease progression
SP: Second primary
DFS: Disease-free survival
DSS: Disease-specific survival
ECOG: Eastern Cooperative Oncology Group
INR: International normalized ratio
PTT: Partial thromboplastin time
GLIM: Global Leadership Initiative on Malnutrition
BW: Body weight
SIB: Simultaneous integrated boost
IMRT: Intensity-modulated radiotherapy
PTV: Planning target volume
VMAT: Volumetric modulated arc therapy
GTV: Gross tumor volume
CTV: Clinical target volume
OARs: Organs at risk
NCI: National Cancer Institute
VFSE: Video fluoroscopic swallowing examination
SAEs: Serious adverse events
RECIST: Response Evaluation Criteria In Solid Tumors
